# Core body temperature responses during competitive sporting events: A narrative review

**DOI:** 10.5114/biolsport.2023.124842

**Published:** 2023-03-03

**Authors:** Gurpreet Singh, Kyle J.M. Bennett, Lee Taylor, Christopher J. Stevens

**Affiliations:** 1 Physical Activity, Sport, and Exercise Research Theme, Faculty of Health, Southern Cross University, Australia; 2 Loughborough University, School of Sport, Exercise and Health Sciences, Loughborough, UK; 3 University of Technology, Sydney (UTS), Human Performance Research Centre, Sydney, Australia; 4 University of Technology Sydney (UTS), Sport & Exercise Discipline Group, Faculty of Health, Sydney, Australia

**Keywords:** Hyperthermia, Physiology, Thermoregulation, Exertional heat illness, Core body temperature

## Abstract

Due to the lack of research in real-world sports competitions, the International Olympic Committee, in 2012, called for data characterising athletes’ sport and event-specific thermal profiles. Studies clearly demonstrate that elite athletes often attain a core body temperature (Tc) ≥ 40°C without heat-related medical issues during competition. However, practitioners, researchers and ethical review boards continue to cite a Tc ≥ 40°C (and lower) as a threshold where athlete health is impacted (an assumption from laboratory studies). Therefore, this narrative review aims to: (i) summarise and review published data on Tc responses during competitive sport and identify key considerations for practitioners; (ii) establish the incidence of athletes experiencing a Tc ≥ 40°C in competitive sport alongside the incidence of heat illness/heat stroke (EHI/EHS) symptoms; and (iii) discuss the evolution of Tc measurement during competition. The Tc response is primarily based on the physical demands of the sport, environmental conditions, competitive level, and athlete disability. In the reviewed research, 11.9% of athletes presented a Tc ≥ 40°C, with only 2.8% of these experiencing EHI/EHS symptoms, whilst a high Tc ≥ 40°C (n = 172; Tc range 40–41.5°C) occurred across a range of sports and environmental conditions (including some temperate environments). Endurance athletes experienced a Tc ≥ 40°C more than intermittent athletes, but EHI/EHS was similar. This review demonstrates that a Tc ≥ 40°C is not a consistently meaningful risk factor of EHI/EHS symptomology in this sample; therefore, Tc monitoring alongside secondary measures (i.e. general cognitive disturbance and gait disruption) should be incorporated to reduce heat-related injuries during competition.

## INTRODUCTION

An athlete’s core body temperature (Tc) increases during strenuous physical activity [1, 2] as locomotion and associated metabolic pathways are thermally ‘inefficient’ at providing energy for muscular contractions [3]. Only ~20–25% of the energy used translates to movement, with the remaining released as heat [4]. The Tc rise is proportional to movement demands [3], and hence, athletes competing in short intermittent activities (e.g. repeated sprinting) or prolonged endurance events (e.g. long-distance running) can both experience a high Tc [3]. In environments where the air temperature and mean radiant temperature are lower than the athlete’s skin temperature (i.e. negative heat gradient), thermoregulatory mechanisms (i.e. cutaneous vasodilation, peripheral blood flow and sweating) and changes in behaviour effectively dissipate excess heat to balance heat gain with heat loss [5]. These mechanisms transfer heat from the core towards the skin surface, where the heat dissipates into the environment via evaporation (predominately), convection, radiation, and conduction [5].

While thermoregulatory mechanisms effectively regulate Tc in cool environments (e.g. heat production does not overwhelm heat dissipation), athletes often compete in hot [6, 7] and/or humid conditions [8, 9], where the capacity for heat exchange decreases [3]. Consequently, if heat production exceeds the body’s ability to dissipate it, an athlete’s Tc will increase [10], often resulting in a reduction in pace or power output [11, 12]. This Tc rise can reduce performance, and in some scenarios, exertional heat illness/heat stroke (EHI/EHS) may ensue [13, 14]. Risk of EHI/EHS is an increasing concern [1], given major-sporting events are increasingly hosted in hot climates due to continued globalisation of sport alongside climate change [12].

**FIG. 1:**
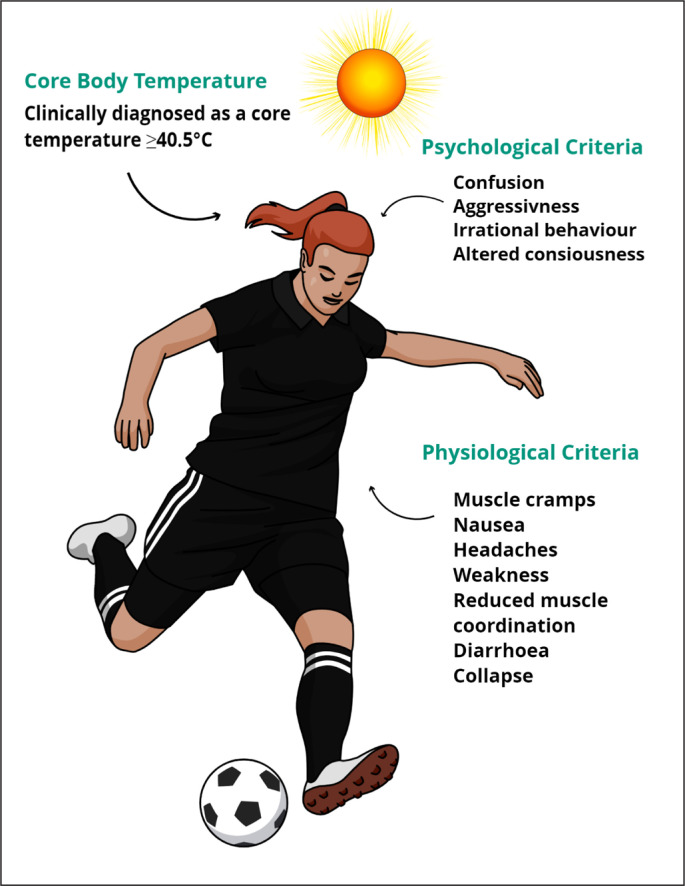
Symptoms of Exertional Heat Illness/Heat Stroke. Note. The information illustrated in this figure was extracted from *Consensus Recommendations on Training and Competing in the Heat and National Athletic Trainers’ Association Position Statement: Exertional Heat Illnesses* [1,75].

In response to these concerns and the lack of research in competitive sport, the International Olympic Committee (IOC) called for research to better characterise sport and event-specific thermal profiles of Olympic and international level athletes competing in the heat [1, 15]. Recent advancements in ingestible telemetric sensor technology allow continuous Tc measurements (with data logging) during an event without the need for any other equipment to be carried by the athlete [16, 17]. Harnessing these technological advancements and thus meeting the IOC call, data now demonstrates that elite athletes can and often attain a Tc ≥ 40°C (a recognised criterion in diagnosing heat illnesses; see Figure 1) without heat-related medical issues arising during competition.

Although the belief that a Tc ≥ 40°C results in health and performance impairments have been heavily criticised [18, 19], practitioners, researchers and ethical review boards continue to mistakenly cite a Tc ≥ 40°C (and lower) as a threshold where athlete health is negatively impacted, which is an assumption derived from laboratory studies [19]. Importantly, practitioners may have a concerning lack of knowledge in this area, as a recent study demonstrated that some practitioners believed a safe Tc was between 29–45°C [20], which is biologically implausible, thus highlighting a need for a resource characterising the Tc profiles of athletes in competitive sport. Nevertheless, studies measuring Tc responses during sports have not yet been collated into a single resource, which could assist practitioners in contextualising the Tc responses across various sports and environments to inform their athletes’ physical preparation and performance plans for upcoming competitions.

Therefore, this narrative review aims to: (i) summarise and review the data published on Tc responses during competitive sport and identify key considerations for practitioners; (ii) establish the incidence of athletes experiencing a Tc ≥ 40°C in competitive sport alongside the incidence of EHI/EHS symptoms; and (iii) discuss the evolution of Tc measurement during athletic competition. A narrative review was chosen since this is a review of descriptive studies [21], with a focus on providing an accessible resource to benefit practitioners, athletes and scientists.

## CORE BODY TEMPERATURE RESPONSES

### Summary of the data

This review included studies that measured athletes’ rectal or gastrointestinal Tc during a competitive sporting event, including events organised by researchers that followed the same regulations used in competition. Athletes competing in all environmental conditions were included (see Table 1). Articles were excluded if they measured Tc within a laboratory environment or during training sessions. Further, this review did not consider skin and tympanic temperatures valid measures of Tc [22]. A data extraction software tool (WebPlotDigitizer, California, USA) was used to retrieve core body temperature data from figures where necessary, and authors were also contacted to confirm these data if required.

**TABLE 1:** Summary of Studies Investigating the Core Body Temperature Responses to Competitive Sport.

Investigation	Sample, Event	Conditions	Tc	Peak Tc	Tc ≥ 40°C	EHI symptoms	Tc Measurement method
Aughey et al. [23]	Elite males (*n* = 35), Australian Football	Hot T_a_: 24–30°C; RH: 40–86% Cold T_a:_ 12–22°C; RH: 29–60%	Average: 37.3–39.4°C	40.5°C	13	0	CorTemp capsules, 60, 30 & 2 min prior & quarters.
Baillot and Hue [8]	Trained males (*n* = 19), Guade-loupe Half Ironman Triathlon	T_a_: 27.2 ± 0.5°C RH: 80 ± 2% WBGT: 25.4 ± 1.0°C Water temp: 29.5°C	Average B (± SD): 37.1 ± 0.7°C Average 1 (± SD):37.8 ± 0.9°C Average 2 (± SD): 37.8 ± 1.0°C Average 3 (± SD): 38.4 ± 0.7°C	40.1°C	1	0	CorTemp capsules, Before & after swim, cycle, run phase
Baillot et al. [24]	Gender not reported (*n* = 20), Trail des Châteaux	T_a_: 25.7 ± 0.6°C RH: 78% WBGT: 23.6 ± 0.9°C	Start mean (± SD): 37.4 ± 0.9°C 11 km mean (± SD): 38.5 ± 1.0°C End mean (± SD): 38.3 ± 0.9°C	39.9°C	0	0	CorTemp capsules, Start, 11^th^ km, end
Bergeron et al. [25]	Youth Boys (*n* = 8), Tennis National Youth Championships	WB GT 30.3 ± 2.5°C	Singles average B (± SD): 37.3 ± 0.3°C Average 1 (± SD): 38.6 ± 0.3°C Average 2 (± SD): 38.6 ± 0.3°C Average 3 (± SD): 38.6 ± 0.2°C Doubles average B (± SD): 38.0 ± 0.4°C Average 1 (± SD): 38.3 ± 0.4°C Average 2 (± SD): 38.5 ± 0.5°C Average 3 (± SD): 38.3 ± 0.5°C	N/a	0	0	CorTemp capsules, Changeovers & after matches
Duffield et al. [31]	Elite males (*n* = 10), Australian Football	T_a_: 29.5 ± 1.3°C RH: 64.9 ± 16.7% WBGT: 27.6 ± 2.3°C	Average B (± SD): 37.2 ± 0.4°C Average 1 (± SD): 37.8 ± 0.5°C Average 2 (± SD): 39.0 ± 0.8°C Average 3 (± SD): 39.1 ± 0.7°C Average 4 (± SD): 39.2 ± 0.9°C Average 5 (± SD): 39.2 ± 0.6°C	N/a	1	0	VitalSense capsules, Before match & quarter breaks
Duffield et al. [9]	Elite males (*n* = 7), A-League Matches	T_a_: 27 ± 2°C RH: 80 ± 10% WBGT: 26 ± 2°C	*Control Group Average* *(± SD)* 1: 38.5 ± 0.3°C 2: 39.9 ± 0.4°C 3: 39.1 ± 0.3°C 4: 39.7 ± 0.8°C *Cooling Group Average* *(± SD)* 1: 28.5 ± 0.2°C 2: 39.7 ± 0.4°C 3: 38.7 ± 0.3°C 4: 39.6 ± 0.6°C	N/a	0	0	VitalSense capsules, Before match, before & after half time
Edwards and Clark [32]	Recreational males (R; *n* = 8), Professional males (P; *n* = 7), Football (soccer)	R T_a_:16°C; RH 47% P T_a_ :19°C; RH 53%	Average 1^st^ R (± SD): 38.5 ± 0.6°C Average 1^st^ P (± SD): 38.1 ± 0.5°C Average 2^nd^ R (± SD): 39.3 ± 0.5°C Average 2^nd^ P (± SD): 38.8 ± 0.5°C	N/a	0	0	CorTemp capsules, 10 min intervals
Fenemor et al. [6]	Elite males (*n* = 11), Oceania Rugby Sevens tournament	Game 1 Ta: 31.3°C RH 71%; Game 2 Ta_:_ 29.0°C, RH: 73%; Game 3 T_a:_ 30.4°C RH: 73%; Game 4 T_a:_ 29.9°C, RH: 75%; Game 5 T_a:_ 26.0°C RH: 81%	N/a	N/a	1	1	e-Celsius capsules, 30 s intervals
Griggs et al. [33]	SCI (*n* = 10) and Non-SCI (*n* = 7), Wheelchair Rugby	T_a_: 18.4–20.9°C RH: 31.1–45.1%	Average SCI (± SD): 37.6 ± 0.4–39.3 ± 0.5°C. Average non-SCI (± SD): 38.1 ± 0.3–38.8 ± 0.3°C	39.3°C	0	0	CorTemp capsules, End of quarters
Henderson et al. [34]	Elite females (*n* = 12) World Rugby 7’s	G1 WBGT: 19.0–19.6°C, G2 WBGT: 18.9–20.0°C G3 WBGT: 18.9–20.1°C	Median (range) G1: 38.4 (37.7–39.1°C) G2: 38.7 (37.9–39.3°C) G3: 38.6 (38.2–39.6°C)	39.9°C	0	0	e-Celsius capsules, 30 s intervals
Hornery et al. [35]	Professional males (*n* = 6), Australian Tennis Circuits	Hard T_a_: 32.0 ± 4.5°C Hard RH: 38.0 ± 14% Clay T_a_: 25.4 ± 3.8°C; Clay RH: 32.0 ± 5%	Average hard (± SD): 38.9 ± 0.3°C Average clay (± SD): 38.5 ± 0.6°C	Hard: 39.3°C Clay: 39.5°C	0	0	Fitsense capsules, Breaks
Hue et al. [36]	Trained males (*n* = 8) Trained female (*n* = 1), Gwadarun	T_a_: 30.0 ± 2.4°C RH: 82 ± 4%	Mean (± SD): 38.5 ± 0.2°C	38.3–38.7°C	0	0	CorTemp capsules, Before and finish
Hue et al. [37]	Elite subjects (*n* = 5), Surfski Ocean Ze Caribbean Race	T_a_: 36.8 ± 2.4°C RH: 68 ± 3.0%	Before mean (± SD): 37.1 ± 0.4°C After mean (± SD): 38.5 ± 0.3°C	38.9°C	0	0	CorTemp capsules, Before and after
Hue et al., [38]	Elite subjects (*n* = 8), Surfski Ocean Racing World Cup	T_a_: 35.9 ± 2.8°C RH: 64 ± 4.0%	Before mean (± SD): 36.7 ± 1.7°C After mean (± SD): 38.1 ± 1.1°C	40°C	1^1^	0	CorTemp capsules, Before and after
Hue et al. [39]	Elite males (*n* = 5) Elite females *(n* = 3), Open Swimming French National Cup	WBGT: 30.0 ± 2.1°C Water temp: 28.1 ± 0.0°C	Mean (± SD): 38.3 ± 0.4°C	N/a	0	0	CorTemp capsules, At 2 km intervals
Høiseth et al. [40]	Trained Males (*n* = 42) Females (*n* = 9), Norseman Xtreme Triathlon	T_a_ = 8.6–15.6°C	Range 36.5–39.4°C	39.4	0	0	e-Celsius capsule, 2 min intervals
Knetchtle et al. [41]	Trained Male *(n* = 1), Ice Mile	Ta: 0°C WS: 2.19 km/h Water temp: 4.8°C	Start mean Tc (± SD) 37.7 ± 0.2°C Range: 36.9–38.3°C Finish mean Tc (± SD) 37.5°C ± 0.6°C Range: 35.9–38.5°C	38.6°C	0	0	Endotherm rectal thermometer,
Knetchle et al. [42]	Elite Males (*n* = 2), Ice swim	T_a_: 0.8°C Humidity: 76% WS: 4 m/s Water temp: 4.3°C	Swimmer 1 start: 37.2°C Swimmer 1 finish: N/a Swimmer 2 start: 37.0°C Swimmer 2 finish: 32.0°C	N/a	0	0	Rectal thermometer, At the start and end of the race
Laursen et al. [43]	Trained males (*n* = 10), Ironman Triathlon	T_a_: 23.3 ± 1.9°C (range: 19–26°C) RH: 60 ± 14% (range: 44–87%) Water temp: 19.5°C	Average (± SD): 38.1 ± 0.3°C	40.5°C	1	0	CorTemp capsules, Before, after swim, cycle, run finish
Lee et al. [44]	Male soldiers (*n* = 31), Singapore Half Army Marathon	Average (range) T_d_: 26.4 (26.1–27.3°C) RH: 81% (79–82%) T_w_ 23.9 (23.7–24.4°C)	Peak average: 39.8 ± 0.5°C Range: 38.5–40.7°C	40.9°C	10	1	Vitalsense & CorTemp capsules,15 s intervals
Lucas et al. [45]	Males (*n* = 7) and females (*n* = 5), Southern Traverse Adventure Race	T_a_ Day 1: 5.2–22.3°C T_a_ Day 2: 11.5–22.2°C T_a_ Day 3: 7.1–11.4°C T_a_ Day 4: 4.4–13.0°C T_a_ Day 5: 2.6–16.8°C	Range: 36.0–39.2°C	N/a	0	0	CorTemp capsules, 1 min intervals
Maron et al. [46]	Trained males (*n* = 2), Santa Barbara Marathons	T_d_: 17.9–21.7°C; T_w_ :13.2–17.4°C	Final Tc Athlete 1: 39.8–41.7°C Final Tc Athlete 2: 39.3–39.9°C	41.9°C	2	0	Rectal Thermometer, 9 min intervals
Mohr et al. [47]	Elite males (*n* = 17), Football (soccer)	T_a_: 21°C, RH: 55% (mod) T_a_: 43°C, RH: 12% (hot)	Average mod 1^st^ (± SD): 38.7 ± 0.2°C, Average mod 2^nd^ (± SD): 38.3°C ± 0.1 Average hot 1^st^ (± SD): 39.6 ± 0.1°C Average hot 2^nd^ (± SD): 39.6°C ± 0.1	N/a	0	0	Phillips rectal thermometer, Half time breaks
Morante and Brotherhood [48]	Males (*n* = 19) And females (*n* = 6), Semi-pro (*n* = 13), Recreational (*n* = 12), Tennis	T_a_: 23.3 ± 7.1°C -26.9 ± 6.4°C WBGT: 20.9 ± 6.2°C -24.4 ± 4.9°C	Range male (± SD): 38.4 ± 0.4°C -38.5 ± 0.48°C Range female (± SD): 38.2 ± 0.3°C-38.4 ± 0.3°C	N/a	0	0	YSI rectal thermistor, 1 min intervals
Morante and Brotherhood [49]	Males (*n* = 19) and females (*n* = 6), Tennis	T_a_: 25 ± 5.4°C (range: 14.5–38.4°C); RH: 50.7 ± 14.3% (range: 21.8–73.7%) WBGT: 22.5 ± 4.3°C (range: 13.5–29.2)	Average (± SD): 38.45 ± 0.36°C Range: 37.43 to 39.98°C	N/a	0	0	YSI rectal thermistor, 1 min intervals
Özgünen et al. [50]	Semi-pro males (*n* = 11), Football (soccer)	G1 T_a_: 34 ± 1°C; RH: 38 ± 2% G2 T_a_: 36 ± 0°C; RH: 61 ± 1%	G1 range: 37.6 ± 0.3°C -39.1 ± 0.4°C G2 range: 37.7 ± 0.4°C -39.6 ± 0.3 1°C	40.2°C	2	0	VitalSense capsules, 10 min intervals
Périard et al. [51]	Males (*n* = 12), ITF Tennis	Cool T_a_: 21.8 ± 0.1°C, Cool RH: 72.3 ± 3.2% Hot T_a_: 36.8 ± 1.5°C, Hot RH: 36.1 ± 11.3%	Average cool (± SD)*:* 38.7 ± 0.2°C Average hot (± SD)*:* 39.4 ± 0.5°C	N/a	0	0	VitalSense capsules, Rest Breaks
Périard et al. [52]	Elite males (*n* = 14), Tour Down Under	T_d_: 23–37°C RH: 19–72% WBGT: 21–29°C	Mean: 38.2–38.5°C	40.2	3	0	e-Celsius capsules, 30 s intervals
Pugh et al. [53]	Gender not reported (*n* = 77), Marathon	T_a_: 22.0–23.5°C; RH: 52–58%; T_w_: 15.5–17.5°C	Range: 36.7–41.1°C	41.1°C	7	4	Rectal thermometer, At the end of the race
Racinais et al. [7]	Elite males & females (*n =* 40), UCI Road World Championships	T_a_: 36.9°C ± 2.8°C; RH: 24.6% ± 15.6% WBGT: 27.1 ± 2.4°C	Average male TTT (± SD): 39.2 ± 0.2°C Average male ITT (± SD): 39.8 ± 0.4°C Average male RR (± SD): 39.6 ± 0.2°C Average female TTT (± SD): 40.8 ± 0.7°C Average female ITT (± SD): 39.9 ± 0.5°C Average female RR (± SD): 39.1 ± 0.4°C	41.5°C	10	0	e-Celsius capsules, 30 s intervals
Racinais et al. [12]	Elite males (*n =* 39) and females (*n =* 17), IAAF World Athletics Championships	T_a_: 29.3°C ± 0.5°C -32.7 ± 0.2°C; RH: 46.3% ± 1.0 -80.6% ± 1.1% WBGT: 23.5°C ± 0.5°C -30.6°C ± 0.3°C	Average pre (± SD): 37.7 ± 0.3°C Average peak post (± SD): 39.6 ± 0.6°C	41.1°C	16	9	e-Celsius capsules, 30 s intervals
Ross et al. [54]	Elite males’ cyclists Tour of Gippsland (*n* = 5) Tour of Geelong *(n* = 5)	T_a_ :15.8°C ± 1.4°C RH: 54 ± 12% T_a_:13.2°C ± 2.1°C RH: 80 ± 8%	Peak average (± SD): 38.9°C ± 0.7°C Peak average (± SD: 39.3°C ± 0.4°C	N/a	0	0	CorTemp capsules, 30 s intervals
Rüst et al. [55]	Trained Males (*n* = 1), Ice Mile Swim	Ta: 9.5 – 11.3°C Water: 9.8–1–10.0°C	Range: 36.0–38.1°C	38.1	0	0	Endotherm rectal thermometer,
Singh et al. [56]	Males (*n* = 5) Females (*n* = 7) Trail Run	Stage 1 T_a_: 11.5–21.7°C, RH: 63–95%; Stage 2 T_a_: 12.4–22.8°C, RH: 54–97%; Stage 3 T_a_: 12.1–21.2°C, RH: 64–96%	Average (± SD): 38.2 ± 0.4°C	40.2°C	2	0	CorTemp capsules, Continuous
Stay et al. [57]	Elite males (*n* = 38) Cricket	Batting T_a:_ range: 22.4–32.8°C RH range: 35.6–69.8% WBGT: 15.6–31.8°C Fielding T_a_ range: 20.9–34.5°C RH range: 34.9–61.3% WBGT: 17.0–31.4°C	Median Batting (IQR): 38.5°C (37.7 – 39.3°C) Median Fielding (IQR): 38.0°C (37.3–38.7°C)	*Batting* 39.3°C *Fielding* 38.7°C	0	8	CorTemp capsules, Rest breaks
Stephenson et al. [58]	Males (*n* = 17) and females (*n* = 11), Iseo-Franciacorta ITU, Paratriathlon World Cup	Event 1 T_a_: 33°C RH: 41% Water temp: 27°C Event 2 T_a_: 33°C RH: 35% Water temp: 25°C	Average acclimatised: 39.78 ± 0.55°C Average non-acclimatised: 39.22 ± 0.41°C Average wet suits: 38.45 ± 0.34°C Average no wet suits: 38.03 ± 0.35°C	N/a	8	16	e-Celsius capsules, 30 s intervals
Stevens et al. [59]	Elite/pre-elite males (*n* = 5) and females (*n* = 9), Commonwealth Games & International Athletics Association Federation Racewalking	R1 T_a_: 25°C, RH: 74% R2 T_a_: 19°C, RH: 34% R3 T_a_: 29°C, RH: 47% R4 T_a_: 23°C, RH: 72%	Average R1 (± SD): 39.70 ± 1.04°C Average R2 (± SD): 39.37 ± 0.67°C Average R3 (± SD): 39.31 ± 0.67°C Average R4 (± SD): 38.95 ± 0.56°C	41.2°C	12	2	e-Celsius capsules, 10 s intervals
Taylor et al. [60]	Elite males (*n* = 17), World Rugby 7’s	Singapore WBGT range: 21.4–27.0°C London WBGT range: 13.8–22.3°C	Singapore G1 range: 36.80–39.10°C Singapore G2 range: 36.40–39.50°C Singapore G3 range: 37.30–38. 50°C London G1 range: 36.00–39.20°C London G2 range: 37.00–39.50°C	N/a	0	0	e-Celsius capsules, 30 s intervals
Tippet et al. [61]	Professional females (*n* = 7), Tennis	WBGT: 30.3 ± 2.3°C Range: 26.1 – 31.9°C	Average Tc (± SD): 38.65°C ± 0.20°C Average Peak (± SD): 39.13 ± 0.20°C	N/a	0	0	CorTemp capsules, 5 min prior, warmup, rests
Valentino et al. [62]	Males (*n* = 23) and females (*n* = 7), Western States Endurance Run	T_a_ 23.6 ± 6.3°C RH: 38.2 ± 16.0%	Average finishers (± SD): 38.2 ± 0.4°C Non-finishers (± SD): 38.2 ± 0.4°C	39.4°C	0	0	CorTemp capsules, 18, 90, 126 km
Veltmeijer et al. [63]	Males (*n =* 111) and Females (*n =* 116), Seven Hill Run	T_d_: 10.5°C RH: 87% WBGT: 11°C	Average start (± SD): 37.8 ± 0.4°C Average finish (± SD): 39.2 ± 0.7°C	N/a	31	0	CorTemp capsules, 1 hour and 15 s before & after the event
Veltmeijer et al. [64]	Elite males (*n* = 6), Wheelchair Tennis	T_d_: 21.2–24.8°C RH: 51.8–61.4% WBGT:17.9– 20.0°C	*Average Tc Increase* (± SD) Injured + 0.6 ± 0.11°C; Non-injured + 0.3 ± 0.1°C	N/a	0	0	CorTemp capsules, 20 s intervals
Wyndham and Strydom [65]	Males (*n* = 31), Sugar Marathons	Event 1 T_a_: 14.8–17.0°C RH: 81–96% Event 2 T_a_: 9.1–16.9°C RH: 29–82%	Peak event 1: 105.1°F (40.8°C) Peak event 2: 105.6°F (40.6°C)	105.6°F (40.8°C)	1	0	Rectal Thermometer, End of event
Yeargin et al. [66]	Youth males (*n* = 16), American Football	Game 1 WBGT 22.0°C Game 2 WBGT: 31.0°C	Peak average game 1: 38.6°C Peak average game 2: 38.7°C	> 39.0°C	0	0	CorTemp capsules, Timeouts
Bongers et al. [26]	Trained males (*n* = 195) and females (*n =* 180), Seven Hills Run, Nijmegen	T_a_ 8–12°C RH: 80–95%	Average (± SD): 39.2°C ± 0.7°C	N/a	M:12% F: 10%	0	CorTemp capsules, Before & after the event
Byrne et al. [27]	Male soldiers (*n* = 18), Singapore Half Army Marathon	T_a_: 27.2 ± 1.0°C RH: 87 ± 5% T_w_: 25.9 ± 0.3°C WBGT: 26.0–29.2°C	Average (range) 30 min: 39.2 ± 0.3°C (38.7–39.8°C) Average (range) 60 min: 39.6 ± 0.6°C (38.5–40.6°C) Average (range) 90 min: 39.7 ± 0.7°C (38.3–41.3°C) Average (range) final 39.9 ± 0.8°C (38.3–41.7°C) Peak average (± SD): 40.1 ± 0.7°C	41.7°C	12	0	CorTemp capsules, 15 s intervals
Christensen and Ruhling [28]	Trained female (*n* = 1), Marathon	T_a_: 12.7–27.9°C RH: unavailable	Tc remained between 37.5–40.0°C	40.0°C	1	0	Rectal thermometer, 10 min intervals
Del Coso et al. [29]	Trained males (*n* = 30) and females (*n* = 4), Ironman Triathlon	T_a_: 29 ± 3°C (range: 24–30°C) RH: 73 ± 8% (range 65–85%) Water temp: 19 ± 1°C	Average start (± SD): 37.5 ± 0.6°C Average after (± SD): 38.8 ± 0.7°C	N/a	0	0	CorTemp capsules, Before & after the event
Diversi et al. [30]	Trained Males (*n* = 6) Females (*n* = 3), Six-hour swim	T_a_: 15–25°C Water temp: 15–15.8°C	Mean (± SD) 36.49 ± 0.79	37.29°C	0	0	CorTemp capsules, interval N/a

A summary of 49 studies that measured the Tc response of athletes during competitive sports is presented in Table 1. The studies represent a sample of 1,450 athletes competing in a range of sports, categorised as intermittent (i.e. association football, Australian football, cricket, rugby sevens and tennis) or endurance sports (i.e. cycling, racewalking, running, triathlon, swimming, adventure racing and surf-skiing). The incidence of a Tc ≥ 40°C, EHI/EHS symptoms and sample demographics/study setting (i.e. competitive level, physical ability, sport type, and environmental risk categories) from the 49 studies appear in Table 2. These data will be used to provide further insight into Tc responses of athletes across the demographic/study settings.

**TABLE 2:** Summary of the Sample Demographics and Study Setting

Sample Demographics	*n* (%)	Tc ≥ 40°C (%)	EHI/Heatstroke (%)
**Sport Types**			
Intermittent sports	295 (20.3)	4.4	2.7
*American football*	16 (1.1)	0	0
*Association football (soccer)*	50 (3.4)	6.0	0
*Australian football*	45 (3.1)	20.0	0
*Cricket*	38 (2.6)	0	21.1
*Rugby union*	57 (3.9)	1.8	1.8
*Tennis*	89 (6.1)	0	0
Endurance sports	1155 (79.7)	13.8	2.8
*Adventure racing*	24 (1.7)	8.3	0
*Cycling*	64 (4.4)	20.3	0
*Racewalking*	53 (3.7)	47.2	3.8
*Running*	838 (57.8)	16.6	0.6
*Swimming*	21 (0.9)	0	0
*Surf Skiing*	13 (0.9)	7.7	0
*Triathlon*	142 (9.8)	7.0	11.3

**Environmental Risk Categories**			
Extreme-risk	170 (11.7)	8.8	7.3
High-very high risk	201 (13.9)	18.4	8.0
Moderate-high risk	220 (15.2)	18.2	1.8
Low risk	757 (52.2)	9.6	0
Low-moderate risk	249 (17.2)	4.8	1.6

**Competitive Level**			
Amateur/recreational	20 (1.4)	0	0
Elite	411 (28.3)	15.3	8.8
Trained	901 (62.1)	11.3	0.1
Unknown	102 (7.0)	6.9	3.9
Youth	16 (1.1)	0	0

**Physical Ability**			
Able-bodied athletes	1,399 (96.5)	11.7	1.8
Para-athletes	51 (3.5)	15.7	31.4

*Note*. The WBGT risk categories included; low (WBGT < 20°C), moderate-high (WBGT 21–25°C), high-very high (WBGT 26–29°C) and extreme (WBGT ≥ 30°C) [67]. While the ambient condition risk categories included; low (Ta 15–20°C), low-moderate (Ta 21–25°C; relative humidity [RH] > 70%), moderate-high (Ta 26–29°C; RH > 60%), high-very high (Ta 31–35°C; RH > 50%) and extreme (Ta ≥ 36°C; RH > 30%) [67]. The competitive level was based on how the authors reported participants in the original investigations. *Key. n* (participant number), Tc ≥ 40°C (incidence of core body temperature ≥ 40°C) and EHI/EHS (number of individuals reported with at least one symptom of exertional heat illness/heatstroke).

### Considerations for practitioners and athletes

#### Physical demands of the sport

Athletes competing in endurance sports had a higher incidence of a Tc ≥ 40°C (13.8%) than athletes in intermittent sports (4.4%). However, the prevalence of EHI/EHS symptoms was similar between both (2.8 vs. 2.7%). Notably, six endurance studies [12, 44, 52, 53, 58, 59] reported at least one symptom of EHI/EHS compared to two for intermittent sports (i.e. cricket and Rugby sevens) [6, 57], but it is unclear whether these symptoms occurred with the co-presence of a Tc ≥ 40°C in some studies.

Evidently, intermittent and endurance sports have different physical demands. Intermittent sports require repeated efforts (including supra-maximal efforts) with short and often incomplete recovery periods [68], whilst endurance athletes typically work at submaximal intensities continuously for long durations with limited or no recovery [69]. Consequently, the contrasting demands drive differential Tc response kinetics [3], given that the rise in Tc is generally proportional to the oxygen consumption in temperate environments, which will vary with mechanical efficiency or movement economy [5]. Australian Football (AFL) athletes, for instance, experience peak Tc during the quarters (regardless of chronological order) with the highest number of maximal accelerations [23], whilst the Tc of endurance runners gradually increases throughout their event, likely due to the more stable exercise intensity and oxygen consumption [27]. Some endurance events have an intermittent component as well, such as road cycling, which involves periods of drafting and free-wheeling, which decreases power output, energy expenditure, heart rate and overall heat storage [54, 70] or conversely, attacks and stages with ascents can increase power output and heat storage [52]. An interesting comparison between road and time-trial cycling events demonstrated that cyclists experience higher Tc during a time-trial than road race event, despite similar environmental conditions and the road race having a far greater duration [7]. Hence, the higher Tc in the time-trial was caused by a higher mean power output (4.7 ± 0.3 W/kg) than in the road race (2.7 ± 0.4 W/kg) [7], which is evidence that the intensity of an activity is a bigger contributing factor to a Tc rise than duration.

Endurance athletes gradually accumulate heat, with peak Tc’s in the latter stages of their events [26, 27, 46, 63]. For example, the Tc of race walkers [59], marathon [27, 44] and endurance runners [8, 24] and swimmers increased initially and then reached a relative plateau (although likely still increasing minimally when time-averaged) or increased at each measurement interval throughout their race [8, 39]. Endurance athletes typically, but not exclusively, perform a large duration of their events at constant intensities (albeit with periods of attacks and surges, including the well-discussed ‘end spurt’ phenomenon) [71]. This consistent locomotion liberates substantial heat, due to the inefficiencies outlined earlier. Whilst these Tc responses to endurance exercise in the heat are typical, there are exceptions. Triathlon, for example, is a sport where peak Tc was seen post-swim, decreasing thereafter in the cycling and running phases [8, 43]. The evaporative, convective, and radiative heat exchange potentials are more favourable during the run and cycle phases compared to the swim, especially when a wetsuit is worn. However, the Tc of swimmers in other studies initially increased [30, 40–42, 55] and then began to decrease approximately 20 minutes into their swim [55]. Interestingly, these swimmers presented a lower Tc at the end of their event, likely due to the cold-water temperatures these swimmers were competing in [30, 40–42, 55]. As such, the variation in Tc responses makes it difficult to simply define a Tc threshold that can affect endurance performance, but endurance athletes that maintain high intensities for a medium-long duration, without recovery opportunities, have an increased likelihood of experiencing a Tc ≥ 40°C.

Core body temperature does not necessarily accumulate across an intermittent event, due to the variability in the physical demands within and between these sports. Professional soccer players can perform 459–856 m of high-speed running within a match [72], and the variability of high-speed running appears to influence the Tc response. Indeed, the peak Tc of soccer players competing in the heat were similar after each half, despite a ~7% reduction in total distance and ~26% reduction in high-speed running in the second half [47]. The players likely adopted a pacing strategy to reduce the number of high speed-efforts in the second half to avoid excessive fatigue in the heat, which is a common strategy by professional soccer players (as observed during the 2014 FIFA World Cup) [73]. Rugby sevens is another intermittent sport played at very high intensities [74]. Yet only one Rugby sevens athlete experienced a Tc ≥ 40°C and presented at least one symptom of EHI/EHS [6] across 13 matches in a range of conditions (WBGT range 18.9°C-31.3°C) [34, 60] and ambient temperature ranges (26.0–31.3°C) [6]. Since a Rugby sevens match is considerably shorter than most sports (only 14-minutes), the short duration combined with breaks in play and substitutions may have precluded the athletes’ Tc from exceeding 40°C. Based on the available research, cricket was the only intermittent sport where multiple athletes reported symptoms of EHI/EHS [57]. Cricket athletes (e.g. batters, close fielders and the wicket-keeper) wear extra protective clothing, which can hinder evaporative heat loss. The extra clothing, coupled with the high ambient temperatures, may explain the reported symptoms. It is also important to consider that the cricket athletes compete over consecutive days, and the risk of EHI/EHS increases the day after an athlete competes in an environment with a high WBGT [75], which may explain the symptoms reported.

### Environmental risk categories

While athletes experienced a Tc ≥ 40°C in all environmental conditions, the highest incidence of a Tc ≥ 40°C was in the “high-very high risk” (18.4%), followed by the “moderate-high risk” (18.2%), “extreme risk” (8.8%), “low risk” (9.6%) and “low-moderate risk” (4.8%) conditions. Unsurprisingly, environmental condition thresholds incorporating an element of ‘high-risk’ (i.e., “high-very high risk” and “moderate-high risk”) had the highest incidence of Tc ≥ 40°C since the thermal gradient between the skin and environment decreases in hot climates (further compounded by the co-presence of high ambient water vapour pressure), thereby compromising heat loss [3].

Direct comparisons between events in different environmental risk categories further demonstrated that athletes’ Tc were generally greater in higher-risk categories. For example, 37.1% of Australian Football athletes in one study experienced a Tc ≥ 40°C during matches played in “high-very high risk” conditions compared to zero in low-moderate risk conditions [23]. Similarly, soccer [47] and tennis [51] athletes’ experienced a significantly higher Tc during matches played in “extreme risk” conditions than in “low-moderate risk” environments. However, tennis athletes in another study did not present a significantly different peak Tc between matches played in “high-very high risk” and “low-moderate risk” conditions [35]. Athletes competing in the “high-very high risk” environmental conditions had significantly longer rest periods between points (25.1 ± 4.3 vs. 17.2 ± 3.3 seconds), which may have attenuated the rise in Tc [35]. Collectively, these studies indicate that athletes competing in higher risk environments can experience a high Tc, even in sports of an intermittent nature. This may introduce a problem for athletes expecting cool conditions at a sporting event but are surprised by hot conditions.

Athletes in “extreme risk” conditions had a lower incidence of a Tc ≥ 40°C than in other high risk environments (i.e. “high-very high risk” and “moderate-high risk” conditions [47, 50–52, 57, 61, 66]. Often amateur or recreational events are cancelled in “extreme risk” conditions, and as such, fewer athletes were competing in these conditions, which likely explains the low incidence of a Tc ≥ 40°C. Elite athletes competing in “extreme risk” environments can also change their behaviour to reduce thermal strain [12, 47, 51]. This was highlighted by research on soccer players, with a 26% decline in high-intensity running distances during a match played in “extreme risk” conditions than in a “low-moderate risk” environment [47]. Tennis athletes competing in “extreme risk” conditions also had a significantly lower effective playing percentage (i.e. time competing on the court) than in the cooler conditions [51]. Elite racewalkers at the 2019 Doha World Athletics Championships had 12% ± 7% slower event times compared to their personal bests [12], likely due to reductions in pace to alleviate thermal strain. Most sports in “extreme risk” conditions were intermittent, which likely provided athletes with rest periods and cooling opportunities (and some sports implement such changes when playing in these environments due to their heat policies). Endurance athletes in “extreme risk” conditions were elite race walkers, marathon runners, ski-surfers or simmers who were heat acclimatised and implemented mid-cooling strategies during their events, which may explain the low incidence of a Tc ≥ 40°C [8, 11, 12, 36–39]. Accordingly, changes in behaviour coupled with the intermittent nature and cooling strategies of the sports played in “extreme risk” conditions may explain the low incidence of a Tc ≥ 40°C. Despite this, 6.8% of cricket athletes competing in these environments reported at least one symptom of EHI/EHS without a Tc ≥ 40°C [57]. This may be explained by cricket athletes wearing extra protective equipment, as mentioned previously.

An interesting finding was that athletes competing in “low risk” [26, 63, 65] and “low-moderate risk” [23, 43, 46, 53, 56] environments could also experience a Tc ≥ 40°C. In particular, 22% [26] and 15% [63] of athletes included in mass participation marathon events experienced a Tc ≥ 40°C in ambient temperatures < 12°C [26, 63]. This may be explained by conditions of high relative humidity (> 80%) that likely contributed to the high incidence of a Tc ≥ 40°C, since the water vapour gradient between the skin and environment reduces in humid climates, impeding the evaporative cooling capacity [3]. Collectively, these observations suggest that athletes can experience a remarkably high Tc in a range of environmental conditions, including climates categorised as “low risk”, with high humidity; therefore, practitioners should be aware of not only the ambient temperature but also the relative humidity of the environment.

### Competitive level

Elite and trained athletes had the highest incidence of a Tc ≥ 40°C (see Table 2). Interestingly, elite athletes had a higher prevalence of at least one symptom of EHI/EHS (8.8%) than trained athletes (0.1%). No amateur/recreational or youth athletes experienced a Tc ≥ 40°C or EHI/EHS symptom. Elite and trained athletes are more likely to be acclimatised to the heat, possessing heightened plasma volume expansion and earlier onset of sweating, resulting in enhanced thermoregulation (i.e. lower peak Tc) [76]. However, elite athletes are more likely to push themselves beyond their limits with very high motivation to perform well in their events and endure a high Tc, if necessary, despite their enhanced thermoregulatory capacity than their lesser-trained counterparts. Elite athletes also have a higher tolerance to heat sensation/pain than non-athletes [77], which may allow them to push themselves beyond their limits.

Several acclimatised endurance athletes commonly experienced a Tc ≥ 40°C [7, 46, 59]. For example, two elite female cyclists experienced a Tc > 41°C without experiencing any symptoms of EHI/EHS [7]. These cyclists participated in a 9-day heat acclimation programme before their event [7], suggesting that this training helped the athletes tolerate a high Tc. In a separate study, elite racewalkers with extensive heat acclimation training often presented a Tc ≥ 40°C, but two athletes reported at least one symptom of EHI/EHS after the event [59]. Racewalkers in another study also reported EHI/EHS symptoms, but the incidence of a Tc ≥ 40°C was low, likely due to the low radiant heat load imposed during the night races at the 2019 IAAF World Championships, reduction in pace and extensive use of mid-cooling strategies [11]. The race-walking competitions were at major sporting events, and hence the athletes were highly motivated to win and therefore tolerated the high Tc during competition.

Athletes who experienced a Tc ≥ 40°C were often the fastest competitors in their events [43, 46, 53] or medal recipients [7, 59]. It appears that elite athletes experiencing a Tc ≥ 40°C did not have impaired health or performance during real-world sporting competitions, likely due to being heat acclimatised from their training and their perceptual tolerance to the heat. This finding raises an ethical dilemma for medical practitioners monitoring real-time Tc during competition to reduce the risk of EHI/EHS, because it is uncertain whether the high Tc observed will result in health impairments. For example, if a marathon runner presents a Tc ≥ 40.5°C, with 1–2 km left, medical practitioners must decide whether to stop the athlete, which may deny them a chance of winning a medal or let them continue, potentially leading to EHI/EHS [16]. Indeed, as discussed throughout this review, athletes do not necessarily present EHI/EHS symptoms when experiencing a Tc ≥ 40°C, and therefore, it is unclear exactly what temperature might be used as a cut-off point in this situation. Instead, practitioners should ensure that they understand an athlete’s thermal tolerance and know EHI/EHS symptoms during competition. Further, a recent paper by Muniz-Pardos et al. (2021) emphasises the importance of developing a system that allows real-time Tc monitoring but accompanied by a secondary measure such as cognitive/biomechanical assessments (i.e. general cognitive disturbance, gait disruption) to protect athletes from EHI/EHS.

### Athlete disability

Para-athletes presented a greater incidence of a Tc ≥ 40°C and prevalence of at least one symptom of EHI/EHS compared to able-bodied athletes (see Table 2). Athletes with a spinal cord injury often have neural impairments that alter physiological responses that affect thermoregulation [78]. One study highlighted this, where 47.1% of the athletes reported at least one symptom of EHI/EHS while competing in a para-triathlon under “high-very high risk” environmental conditions [58]. Ten athletes also competed in wetsuits [58], which can provide a competitive advantage in the water, but are also designed to prevent heat loss [79]. Consequently, a combination of impaired thermoregulation, high-risk conditions and wetsuit use may have increased the athletes’ thermal stress. Two studies comparing the rise in Tc between spinal cord-injured and non-injured athletes reported spinal cord-injured competitors with a greater Tc rise among spinal-cord injured athletes in wheelchair rugby [33] and tennis matches [64]. Based on these studies, it appears that spinal cord injured athletes, especially when competing in higher-risk conditions for long durations, are at increased risk of EHI/EHS. Therefore, practitioners should ensure such athletes are prepared for these conditions and consider using cooling strategies to attenuate thermal stress caused by impaired thermoregulation.

## EVOLUTION OF CORE BODY TEMPERATURE MEASUREMENT

The measurement of Tc in thermoregulation research during competitive sports has evolved over the last 50 years [80]. Traditionally, researchers would collect Tc using rectal probes when performing stationary exercises or before and after an event (see Table 1) because these probes require a wired connection to a data logging device. Alternatively, athletes would insert a rectal probe before an event, hide the cord within their clothes whilst competing and then connect to a data logger during breaks. The limited data collection opportunities and invasive nature of rectal probes have led researchers to use ingestible telemetric capsules, which wirelessly transmit Tc measurements to a data logger via telemetry [81]. Earlier systems (i.e. CorTemp and VitalSense systems) required athletes to carry a data logger on their person to visualise or store the data. However, this limits data collection opportunities, as the data-logger could add unwanted weight to the athlete, interfere with their technique, and increase the injury risk for athletes in contact sports (plus, carrying data loggers during competition in contact sports is often prohibited for obvious reasons). As a result, researchers could still only collect Tc data during break periods in many events, meaning that the athletes’ peak Tc could not be deduced from the limited data collected. More recently, advancements to ingestible telemetric capsule technology have produced a solution, whereby data can be stored within the capsule itself and transmitted wirelessly to a portable receiver at a later time (i.e. e-Celsius) [80]. This technology has enabled researchers to collect continuous Tc data in situations where the individual is not permitted to carry a data logger with them (i.e. in team-sports and elite competitions/events). Indeed, Tc measurement during competition via telemetry has been performed since 2006 (see Table 1), specifically in situations where a data logger could be attached on an athlete or when Tc measurements could be taken at intervals/breaks in play. To our knowledge, the first published study using the e-Celsius technology during sports competition included in this review was in 2019 [60], and since this time, nine studies have used this to contextualise the thermal demands across different sports [6, 7, 12, 34, 40, 52, 58–60].

## LIMITATIONS

Athletes and practitioners should be aware of the following limitations of this review. First, there was likely a higher incidence of athletes experiencing a Tc ≥ 40°C across the literature, since the limitations of the available technology described above meant that continuous Tc measurements could not be recorded in many of the cited studies. Furthermore, the Tc measurement frequency was inconsistent across the studies, with researchers sampling Tc from anywhere between 10 seconds to 10 minutes or only during breaks in play. Studies with more frequent sampling better profile Tc changes during competition, and studies with less frequent sampling may not capture important fluctuations in Tc between measurements. Therefore, future research should aim to continuously measure Tc throughout the event with a sampling frequency of at least every 1 minute. Additionally, the Tc data presentation across the studies was also inconsistent, with researchers reporting the average, median or Tc range, and the peak Tc was often not clear. Researchers are encouraged to present full data traces of the mean Tc across the event to increase the utility of the work.

Researchers have recommended consuming these capsules at least six hours before the first measurement, allowing them to transit through the stomach and into the intestinal tract to avoid coming into direct contact with ingested liquids [80, 81]. However, in some situations, athletes were unable to ingest a capsule within this timeframe; for example, cricket athletes in one study ingested telemetric capsules three hours before the first session each day, and as such, the initial Tc measurements on each day of the study may be inaccurate [57]. Researchers can instruct athletes to ingest a capsule the night before [82], although the capsules can pass with the first bowel movement the next morning, which was highlighted in a recent cycling study [52], resulting in limited data. One solution to overcome these issues is to insert the capsule into the rectum to measure rectal Tc instead of gastrointestinal Tc, as successfully performed in one of the studies [59].

Finally, several limitations likely exist in reporting EHI/EHS symptoms. Athletes may not have accurately reported EHI/EHS symptoms due to fears that the coaching staff may reduce their playing time. Alternatively, athletes may not have been provided with enough opportunity (or any opportunity) to report these symptoms. Indeed, many studies did not explicitly state that they measured the occurrence of such symptoms. Further, the Tc of the athletes’ that reported EHI/EHS symptoms could not be determined from the reviewed studies.

## CONCLUSIONS

After collating the Tc responses from various sports and environmental conditions, it is evident that athletes commonly experience a Tc ≥ 40°C during real-world competitive events. It appears that the physical demands of the sport, environmental conditions, competitive level, and athlete disability are contributing factors to a high Tc. Despite athletes commonly experiencing a Tc ≥ 40°C, the prevalence of EHI/EHS symptoms was low. Practitioners should also be aware that athletes competing in all environmental conditions can experience a Tc ≥ 40°C, particularly in climates categorised as “high-very high risk” and “moderate-high risk”. When the competitive level was compared, elite and trained athletes commonly had a Tc ≥ 40°C, with elite athletes presenting the highest prevalence of EHI/EHS symptoms. Nevertheless, spinal-cord injured athletes experienced the greatest occurrence of EHI/EHS symptoms due to neurological and physiological impairments that impact thermoregulatory pathways. Collectively, these findings suggest that a Tc ≥ 40°C is not a consistently meaningful risk factor of EHI/EHS in this sample; therefore, Tc measurement alongside secondary measures should be incorporated to safeguard athletes during competition.
